# The Multi-Attribute Method (MAM), An Advanced LC-MS Approach for Protein A Resin Performance and Lifecycle Evaluation

**DOI:** 10.3390/antib15020026

**Published:** 2026-03-23

**Authors:** Jingming Zhang, Matthew Larsen, Timothy Blanc, Babita S. Parekh, Ming-Ching Hsieh

**Affiliations:** 1 Analytical Sciences, Eli Lilly and Company, Branchburg, NJ 08876, USA; jingming.zhang@network.lilly.com; 2 Technical Service/Manufacturing Service, Eli Lilly and the Company, Branchburg, NJ 08876, USA; 3 Manufacturing Process Development, Eli Lilly and Company, Branchburg, NJ 08876, USA

**Keywords:** multi-attribute method (MAM), liquid chromatography–mass spectrometry (LC-MS), peptide mapping, Protein A resins, post-translational modifications (PTMs), host cell proteins (HCPs), clean-in-place (CIP), resin fouling

## Abstract

Background: Protein A resins are indispensable for monoclonal antibody (mAb) production, yet their condition and performance are traditionally assessed using indirect or qualitative methods. In this study, the multi-attribute method (MAM), previously applied to therapeutic protein characterization, is systematically adapted for the first time as a unified liquid chromatography–mass spectrometry (LC-MS) platform for Protein A resin analysis. Method: Four Cytiva Protein A resins, MabSelect™, MabSelect SuRe™, MabSelect SuRe™ LX, and MabSelect™ PrismA, were evaluated by MAM for resin identity, Protein A ligand integrity, fouling by impurities, and cleaning performance. Results: MAM enables resin-specific peptide fingerprinting and quantitative monitoring of Protein A ligand post-translational modifications (PTMs), including deamidation, isomerization, and fragmentation induced by repeated clean-in-place (CIP) cycles. Comparative analysis of virgin and used resins revealed ligand degradation and fouling despite engineered alkaline stability, with MabSelect™ showing the greatest susceptibility. Importantly, residual monoclonal antibodies (mAbs) and host cell proteins (HCPs) were directly detected and quantified from the resin matrix, providing a molecular-level assessment of resin cleaning effectiveness not achievable with conventional approaches. Conclusions: This work establishes MAM as a novel, sensitive, and comprehensive strategy for Protein A resin lifecycle management, delivering actionable insight for resin selection, cleaning optimization, and downstream process development.

## 1. Introduction

Protein A affinity chromatography is widely used as the primary capture step in the purification of therapeutic monoclonal antibodies, including IgG_1_, IgG_2_, and IgG_4_ subclasses, as well as Fc-fusion proteins, in biopharmaceutical manufacturing [1]. Its use has increased substantially as monoclonal antibodies have become a major class of pharmaceuticals: more than 200 therapeutic antibodies have reached the market, and over 1000 investigational candidates are currently being evaluated [2]. In addition, more than 50 biosimilar therapeutic antibodies have been approved by the U.S. Food and Drug Administration (FDA) [3]. The global therapeutic antibody market was valued at about US$250 billion in 2024 and is projected to double to US$500 billion by 2029, with a compound annual growth rate (CAGR) of 14.5% [4].

Because of its high affinity and selectivity, Protein A chromatography provides greater than 95–98% purity in one step [5,6]. Protein A, a 42 kDa cell wall protein from *Staphylococcus aureus*, is encoded by the *spa* gene and has five immunoglobulin-binding domains (E, D, A, B, and C) [7], each adopting a three-helix bundle. In commercial resins, Protein A is immobilized on a solid support, typically agarose or another polymeric bead matrix, to enable efficient binding and elution of antibodies [8]. This purification process has become the industry standard for the affinity capture step in the manufacturing of monoclonal antibodies and Fc-fusion proteins, owing to its scalability, reproducibility, and established regulatory acceptance. However, the cost of Protein A resin is a major economic factor in monoclonal antibody (mAb) production. For process-scale chromatography in the biopharmaceutical industry, where the diameter of the Protein A column could exceed 100 cm, resin expenditures can exceed $1 million [9]. The high cost stems partly from continuous development aimed at improving immobilization chemistries [10,11] and protein sequence mutations designed to improve caustic stability, thereby minimizing degradation and fouling to extend the usable lifecycle of the resin [12,13].

Due to the high cost of downstream processing, Protein A resins are typically reused for over 200 binding-elution cycles [9,14]. However, as a protein-based ligand, Protein A is prone to degradation after repeated use, highlighting the importance of employing optimized procedures for washing, sanitization, cleaning, and storage [15]. Protein A resin fouling arises primarily from non-specific adsorption of feed stream components, including host cell proteins (HCPs), mAb aggregates and fragments, media constituents, and other impurities that accumulate on and within the bead surface, blocking pores and limiting access to the ligand [14,16,17]. Proteolytic enzymes among the HCPs can further contribute to resin degradation. Physical obstruction by deposited HCPs and media components, together with chemical degradation of the Protein A ligand under harsh cleaning conditions, progressively diminishes resin performance. Standard industrial clean-in-place (CIP) regimes include inter-cycle washing, rigorous post-use cleaning (typically with alkaline agents), sanitization, and controlled storage. Although these procedures are essential to maintain chromatographic performance and limit microbial contamination, they can also shorten resin lifetime by promoting degradation of both the ligand and the underlying matrix [18,19].

The multi-attribute method or monitoring (MAM) is an advanced analytical strategy that combines liquid chromatography (typically reverse-phase) with high-resolution mass spectrometry (HRMS) to simultaneously monitor multiple critical quality attributes (CQAs) within a single assay. The peptide-based MAM workflow is designed to streamline product characterization and quality control by consolidating several conventional assays into one LC-MS platform [20]. For therapeutic antibodies, MAM enables a comprehensive assessment of attributes, such as product identity, fragmentation, disulfide bond integrity, glycosylation profiles, charge variants, deamidation, isomerization, oxidation, and N- and C-terminal modifications in a single analysis. This approach has the potential to replace multiple traditional techniques, including ion exchange chromatography (IEX), size exclusion chromatography (SEC), N-linked glycan analysis via normal-phase (NP) or hydrophilic interaction chromatography (HILIC), hydrophobic interaction chromatography (HIC), and denaturing-capillary gel electrophoresis (CE-SDS).

The concept of MAM was introduced in 2012 [21], with the first peer-reviewed publication appearing in 2015 [22]. Since then, adoption has accelerated through key milestones: the formation of the MAM Consortium in 2016, active engagement by the FDA to integrate MAM into QC environments [23], and the official publication of USP General Chapter 〈1060〉 “Mass Spectrometry-Based Multi-Attribute Method for Therapeutic Proteins” on 1 August 2025 [24]. While initially developed for therapeutic proteins such as monoclonal antibodies (innovator and biosimilar), bispecific antibodies (BsAbs), antibody-drug conjugates (ADCs), and fusion proteins [25], MAM has also been extended to cell and gene therapy products, including characterization of adeno-associated virus (AAV) capsid proteins [26].

In this study, we present the first fully developed application of an LC-MS-based MAM for simultaneously monitoring the chemical and structural integrity of Protein A resins. The method was applied to comprehensively address three critical aspects of resin lifecycle management: (1) confirmation of Protein A ligand identity, (2) identification of fouling- and cleaning-related chemical modifications to inform appropriate CIP strategies, and (3) evaluation of residual mAb and HCPs remaining on used resins.

Historically, Protein A resin fouling and degradation have been investigated using a diverse set of largely orthogonal analytical techniques, including particle size analysis, HPLC, fluorescence spectroscopy, SDS-PAGE, scanning and transmission electron microscopy (SEM, TEM), LC-MS, ATR-FTIR, confocal laser scanning microscopy (CLSM), and Raman spectroscopy [17,27,28]. Residual HCPs have typically been assessed using SELDI-TOF MS, ELISA, or LC-MS [29]. While valuable, these approaches are generally single-purpose, require multiple platforms, and do not provide an integrated, sequence-level assessment of resin identity, fouling chemistry, and residual impurities.

By extending MAM from therapeutic protein characterization to Protein A resins, this work establishes a unified LC-MS-based framework with several advantages over prior approaches. Peptide mapping provides sequence-level confirmation of Protein A ligand identity and direct detection of chemical modifications associated with resin aging and CIP exposure, enabling informed lifecycle management. The method further enables site-specific identification of ligand degradation and simultaneous quantification of residual bound antibody, HCPs, and resin quality attributes in a single LC-MS analysis. In addition, use of 21 CFR Part 11-compliant LC-MS software (for example, Chromeleon^TM^ Version 7.3.2) supports GMP-compatible data integrity, traceability, and regulatory readiness.

## 2. Materials and Experiments

### 2.1. Materials

The therapeutic monoclonal antibodies MAB1 (IgG_4_) and MAB2 (IgG_1_), as well as MAB3 (fusion protein), were manufactured by Eli Lilly and Company. The molecules used in this study are proprietary therapeutic proteins, and their specific identities are withheld to protect intellectual property.

All chemicals are analytical grade or higher, or otherwise specifically described. Acetonitrile (HPLC grade) was purchased from Mallinckrodt Baker, Inc. (J.T. Baker, Phillipsburg, NJ, USA). Trypsin (sequencing grade, TPCK-modified) was obtained from Promega Corporation (Madison, WI, USA). Methanol (HPLC grade), Tris Base, and sodium chloride were purchased from Mallinckrodt Baker, Inc. (J.T. Baker, Phillipsburg, NJ, USA). Acetic acid, phosphoric acid, hydrochloric acid, sodium hydroxide, and 94.9–96% ethanol (non-denatured) were purchased from Sigma-Aldrich (MilliporeSigma, Burlington, MA, USA). Pierce^TM^ trifluoroacetic acid (TFA), sequencing grade, was purchased from Thermo Fisher Scientific (Waltham, MA, USA). For HPLC buffers and solvents, 18 MΩ water from Millipore Milli-Q^®^ Integral 5 Water Purification System, MilliporeSigma (Burlington, MA, USA) was used. Costar^®^ Spin-X^®^ centrifuge tube filters with a 0.45 µm Pore CA membrane were purchased from Corning Incorporated (Salt Lake City, UT, USA). Corning™ Costar™ low-binding plastic microcentrifuge tubes were purchased from Thermo Fisher Scientific (Waltham, MA, USA). All Protein A resins, including MabSelect™, MabSelect SuRe™, MabSelect SuRe™ LX, and MabSelect™ PrismA, as well as the prepacked HiScreen PrismA column, were purchased from Cytiva (Marlborough, MA, USA). All used resins were produced internally following various processing cycles.

### 2.2. Used Resins from Manufacturing Process

MabSelect™, MabSelect SuRe™, and MabSelect SuRe™ LX resins were used for the purification of MAB1, MAB2, and MAB3, respectively. Upon binding, mAbs were eluted with 20 mM acetic acid/5 mM citric acid, 75 mM acetic acid, and 10 mM sodium citrate at pH 3.0, respectively. The corresponding CIP conditions were 0.05 N NaOH with 1 M NaCl for MabSelect™, 0.1 N NaOH and subsequently 1 M acetic acid for MabSelect SuRe™, and 0.05 N NaOH with 1 M NaCl for MabSelect SuRe™ LX. The total number of process cycles was 116, 291, and 112, respectively.

### 2.3. Laboratory Protein A Chromatography Method

MabSelect™ PrismA resin was used in pre-packed Hi-Screen columns (Cytiva, Marlborough, MA, USA) featuring a 10 cm bed height to study different processing conditions and the cleanability (Table 1). In addition, for Condition 5 (Table 1), a borosilicate glass column measuring 1.1 × 10 cm was packed to ensure the height equivalent to a theoretical plate (HETP) and asymmetry values met internal specifications. The columns were equilibrated with 100 mM Tris-HCl Buffer at pH 7 (filtered through a 0.22 µm bottle-top filter with polyethersulfone, or PES, membrane) to establish binding conditions. The Detergent Viral Inactivated (DVI) process intermediate containing MAB1 was filtered using a Millipore Express 0.22 μm PES bottle-top filter prior to loading onto the Protein A column. The chromatography was performed on an ÄKTA Avant 25 system (Cytiva, Marlborough, MA, USA) at ambient temperature, controlled by UNICORN software (v.7.6) with a linear velocity of 275 cm/h and a residence time of 4 min. Following equilibration, the DVI intermediate was applied to the Protein A affinity column at a controlled linear velocity to achieve a consistent residence time, thereby promoting efficient interaction between the Fc region of MAB1 and the immobilized Protein A ligand. Each column was intentionally loaded beyond the conventional resin capacity to maximize utilization of available binding sites during each cycle. Non-binding impurities, including HCPs and residual DNA, were removed through sequential washes: Tris buffer at pH 7 (Wash 1), Tris buffer containing sodium chloride at pH 7 (Wash 2), and Tris buffer at pH 7 again (Wash 3). Bound MAB1 was subsequently eluted using a low-pH solution containing 20 mM acetic acid and 5 mM citric acid at pH 3.5, which effectively disrupted the Fc–Protein A interaction.

Column strip and cleaning were performed according to the study schedule, using various solutions and concentrations as outlined in Table 1. The column was re-equilibrated before subsequent use until the cycling on the resin was complete. All buffers were prepared using raw materials of comparable grade to those employed in GMP manufacturing processes.

### 2.4. Resin Cleanability and Lifecycle Evaluation

The cleanability and lifecycle assessment of Protein A resin were conducted using Hi-Screen prepacked columns containing PrismA resin. Study parameters are detailed in Table 1. Each column underwent 20 chromatography cycles with MAB1, spanning six distinct test conditions in addition to the control (Condition 1). Columns were cleaned with the designated CIP buffers (referenced in Table 1) after every four load and elution cycles for all conditions except Condition 6, in which CIP was carried out following each load and elution cycle.

### 2.5. The Measurement of Dynamic Binding Capacity (DBC)

Dynamic binding capacity (DBC) was assessed by analyzing breakthrough curves with a small-scale column at a constant flow rate (275 cm/h) on an ÄKTA Avant 25 system. The purified drug samples were diluted to representative concentrations (~2–5 mg/mL) using equilibration buffers (50 mM Tris for MAB1 and MAB3, and PBS for MAB2), then continuously applied to Protein A columns that had been equilibrated with Tris or PBS buffer between pH 7–8. UV absorbance at 280 nm was monitored in the column effluent to determine when the target molecule stopped binding to the column and started appearing in the flow-through. Loading ended once the UV signal reached 10% of its maximum, after which washing, elution, and regeneration steps were performed. The breakthrough point, known as QB_10_, is defined as 10% breakthrough of the maximum UV absorbance and calculated using the formula below.

$$DBC=\frac{C_{0}\times (V_{f}-V_{h})}{V_{c}}$$

where *V_f_* is the volume of protein loaded at 10% breakthrough, *V_h_* is the hold up volume of the system, *C*_0_ is the protein concentration of the feed, and *V_c_* is the column volume. DBC is measured in triplicate, and the results are averaged to obtain the final dynamic binding capacity.

The MAM methodology for virgin and used resins is described in Section 2.6.

### 2.6. The Analysis of Protein A Resins by the Multi-Attribute Method (MAM)

The resin (virgin or used) slurry was stored in the original storage solution, 20% ethanol (aqueous). One hundred microliters (100 µL) of virgin or used resin slurry was transferred to a Costar^®^ Spin-X^®^ centrifuge tube filter with a clean low-binding centrifuge tube and then centrifuged at 3000 rpm for 3 min using a Galaxy 20 R refrigerated microcentrifuge (VWR International, Radnor, PA, USA) to remove the storage solution. The dried resin was transferred into a pre-weighed low-binding tube, accurately weighed to determine its dry mass, and then resuspended in 50 mM Tris-HCl buffer (pH 7.5) to obtain a final concentration of 100 mg/mL. An aliquot of 20 µL of MabSelect™ slurry was transferred to a separate tube and incubated with 5 µg of trypsin. In contrast, 10 µL aliquots of MabSelect SuRe™ and MabSelect SuRe™ LX slurries were transferred to separate tubes, and 10 µg of trypsin was added to each preparation. Proteolytic digestion was performed overnight at 37 °C with continuous agitation. The digested solution was transferred to another Spin-X^®^ filter positioned above a new collection low-binding tube and centrifuged using the benchtop centrifuge for 30 s to separate the solution from the resin. The digestion was terminated by adding 5 µL of 20% TFA to the separated solution. A 5 μL aliquot of the digested solution was injected for identity and PTM analysis, while a 50 μL aliquot was used for residual protein (HCP determination).

The resultant peptides were separated using a C18 reverse-phase column (Aeris widepore XB-C18, 200 Å, 3.6 µm, 250 × 2.1 mm, Phenomenex, Torrance, CA, USA) on an Agilent HPLC 1260 series system (Santa Clara, CA, USA) interfaced with an Exploris 480 mass spectrometer (Thermo Fisher Scientific, San Jose, CA, USA) equipped with an electrospray source. Elution of the digested sample for resin identity and PTM analysis was conducted using a gradient from 0% to 39% solvent B (solvent A: 100% H_2_O with 0.05% TFA; solvent B: 100% acetonitrile with 0.04% TFA) over 47.5 min at a flow rate of 0.2 mL/min and a column temperature of 50 °C. For HCP analysis, elution was performed under the same conditions, with an extended gradient over 95 min.

The Exploris 480 mass spectrometer operates with a spray voltage of 3.6 kV, a sheath gas flow rate of 25, an auxiliary gas flow rate of 8, an ion transfer tube temperature of 275 °C, and a vapor temperature of 200 °C. The MS data were acquired by data-dependent acquisition (DDA) as follows: After the survey scan with a positive mass range of *m*/*z* 250–2000, the six most abundant ions were selected for subsequent higher-energy collisional dissociation (HCD) events. The resolution was set at 120,000 for full MS and 15,000 for MS/MS events. 

### 2.7. Mass Spec Data Analysis

The raw LC-MS data were exported from the mass spectrometry computer system and imported into BYOS (Protein Metrics, New York, NY, USA) for analysis using a customized database. For identity and PTM studies, the MS data were searched against a customized database that included sequences of various monoclonal antibodies (mAbs), Protein A, and trypsin. PTMs were identified through MS/MS spectra and quantified using extracted ion chromatogram (XIC) integration. Peptide assignments were reviewed manually to confirm accuracy and PTM localization. In HCP studies, MS DDA data were searched against a protein database containing the Uniprot CHO-K1 database (version 2017), supplemented with sequences for multiple mAbs, Protein A, digestion enzymes, and common contaminants such as keratins. Database searches utilized trypsin as the enzyme, allowing up to two missed cleavages, with full mass tolerance set at 10 ppm and MS/MS mass tolerance at 20 ppm. The HCPs were considered as positively identified when a minimum of two unique peptides, each five or more amino acids in length, were detected. Quantification for each HCP was performed by comparing the peak areas of the three most abundant peptides with those of the spiked protein and expressed in parts per million (ppm). The main mAb product was quantified in a similar manner, with results reported as percentage (%).

## 3. Result and Discussion

Protein A resins are essential affinity media for mAb purification, owing to their high specificity and binding capacity for IgG [15]. They encompass a diverse range of materials, differing in base matrices (e.g., agarose, cross-linked agarose, synthetic polymers, and glass) and ligand formats, including native, recombinant, and engineered Protein A variants.

This study applied the MAM strategy to evaluate four Cytiva Protein A resins, MabSelect^TM^, MabSelect SuRe^TM^, MabSelectSuRe^TM^ LX, and MabSelect^TM^ PrismA, used for manufacturing at Eli Lilly and Company. By focusing on this standardized platform, the study provides a controlled assessment of how analytical MAM strategies can be applied to diverse resin generations within a consistent manufacturing ecosystem. Cytiva’s resins employ recombinant Protein A ligands engineered with a single C-terminal cysteine for site-specific covalent attachment to a highly cross-linked agarose matrix via stable thioether bonds [30]. While the original MabSelect™ ligand shows limited stability under strongly alkaline CIP conditions, MabSelect SuRe™ incorporates an engineered variant with enhanced alkaline resistance while maintaining high IgG Fc-binding affinity. Enhanced alkaline stability in MabSelect SuRe™ was achieved through targeted modification of alkali-labile regions, reducing ligand degradation during repeated CIP cycles [31]. MabSelect SuRe™ LX retains this engineered ligand while offering increased DBC for improved capture from high-titer feeds [32]. MabSelect™ PrismA further combines an engineered Protein A-derived ligand with an optimized high-flow agarose matrix, enhancing DBC, alkaline stability, and process productivity [33].

Application of the MAM strategy to Protein A resins enables simultaneous monitoring of multiple critical quality attributes. These include (i) differentiation of resin types based on characteristic ligand peptide sequences, (ii) assessment of Protein A ligand integrity, including fragmentation and chemical modifications such as isomerization and deamidation, and (iii) detection of residual or fouling-related proteins remaining on the resin after use. This approach parallels established MAM strategies for mAb characterization and provides a powerful analytical framework for resin quality monitoring and lifecycle management.

### 3.1. Multi-Attribute Method (MAM) for Identity Characterization of Protein A Resins

Protein A resins typically appear as white or off-white spherical beads that are virtually indistinguishable by visual inspection. In cGMP manufacturing, where multiple resin types with varying ligands and base matrices are utilized, a robust identification method is essential to prevent mislabeling or cross-contamination. While techniques like Fourier Transform Infrared (FTIR) spectroscopy, ligand density measurement, and binding capacity profiling are common [27], they often lack the specificity required for definitive resin identification. The MAM approach offers a more effective alternative by leveraging the unique amino acid sequences of Protein A ligands. By employing proteolytic digestion (e.g., with trypsin) followed by LC-MS analysis, a distinct peptide profile is generated. This “molecular fingerprint” enables the reliable confirmation of resin identity across different types.

**Figure 1:**
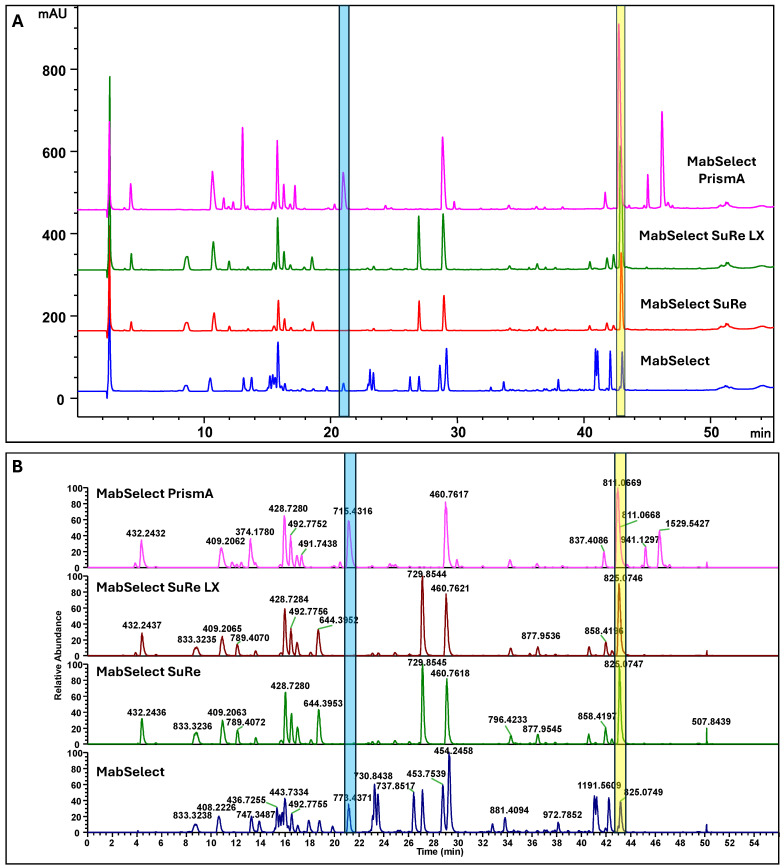
Comparative tryptic peptide profiles of Cytiva Protein A resins. Analytical results include (**A**) HPLC UV traces and (**B**) Base Peak Chromatograms (BPCs) obtained via mass spectrometry. Individual peak labels in the BPC plots indicate specific *m*/*z* values. Shaded color (blue and yellow) blocks identify regions where distinctive peptides co-elute at similar retention times; detailed identification for these regions is provided in Table 2.

Tryptic peptide maps for MabSelect™, MabSelect SuRe™, MabSelect SuRe™ LX, and MabSelect™ PrismA are shown in Figure 1, featuring HPLC UV traces (Figure 1A) and mass spectrometry base peak chromatograms (Figure 1B). While most resins exhibit unique mapping profiles, MabSelect SuRe™ and MabSelect SuRe™ LX produce identical patterns because they utilize the same alkali-stabilized Protein A ligand.

MabSelect™ resin employs a recombinant Protein A ligand derived from the canonical multi-domain architecture (E, D, A, B, and C) of native Protein A, lacking the cell wall anchoring region and thus suitable for chromatographic applications. In contrast, MabSelect SuRe™ and MabSelect SuRe™ LX utilize an engineered Protein A-derived ligand based on repeated Z-domain units, specifically designed to enhance resistance to alkaline degradation during repeated NaOH cleaning cycles [34]. While MabSelect SuRe™ and MabSelect SuRe™ LX share identical ligand sequences, MabSelect SuRe™ LX provides increased dynamic binding capacity, attributed to resin-level optimization that supports higher effective ligand utilization [35]. MabSelect™ PrismA represents the next generation of this platform, incorporating further ligand and matrix optimization to deliver enhanced binding capacity and improved chemical robustness under manufacturing conditions. From an analytical perspective, MabSelect™ generates a larger number of unique tryptic peptides during LC-MS analysis due to the presence of multiple non-identical binding domains, resulting in a more complex peptide-mapping chromatogram with additional peaks (Figure 1). In contrast, MabSelect SuRe™, MabSelect SuRe™ LX, and MabSelect™ PrismA exhibit simpler LC-MS profiles reflecting their repetitive ligand architectures. MabSelect™ PrismA further displays a distinct peptide-mapping profile, consistent with extensive ligand engineering to improve alkaline stability (Figure 1). Although MabSelect SuRe™ and MabSelect SuRe™ LX show similar overall peptide-mapping patterns, MabSelect SuRe™ LX exhibits a higher UV total peak area (TPA), enabling clear analytical differentiation between these two resins.

Mass spectrometry (MS) provides a more comprehensive means of distinguishing among Protein A resins than UV-based peptide mapping, as MS can resolve peptides based on unique mass-to-charge (*m*/*z*) values even when multiple species co-elute at similar retention times. In such cases, UV detection alone lacks sufficient specificity for definitive discrimination. This principle is illustrated in Table 2 and Figure 1 using peptide-mapping data from MabSelect™, MabSelect SuRe™ (including SuRe™ LX), and MabSelect™ PrismA resins.

In the co-elution example highlighted in the blue box, MabSelect™ PrismA and MabSelect™ generated peptide peaks with nearly identical retention times (20.985 min and 21.011 min, respectively). UV-based peptide mapping displayed these features as a single, slightly shifted peak, whereas MS analysis revealed them to be distinct peptides with different *m*/*z* values. A similar phenomenon was observed for peaks within the yellow-boxed region, which differed by approximately 0.3 min in retention time. In this case, the peptide specific to the MabSelect™ PrismA resin was unambiguously identified by its distinct *m*/*z* value of 811.067, a level of differentiation that could not be achieved using UV detection alone.

A Protein A resin identification method can be validated in a QC environment using either LC-only or LC-MS, ensuring consistent selection of the correct resin throughout the purification process. The choice of method should be guided by a balance between regulatory requirements and a risk-based approach that is fit for purpose. While both LC-only and LC-MS methods can be validated [36,37,38,39,40], LC-only methods are generally simpler to implement and validate under ICH Q2(R2) guidelines, making them the preferred option for routine QC analysis. LC-MS, on the other hand, offers enhanced characterization capabilities, including the ability to confirm peak identity and detect subtle structural differences; however, its validation process is more complex and resource-intensive. Beyond basic identity testing, advanced analytical strategies, such as peptide mapping, can be employed to monitor structural integrity and chemical modifications of Protein A resin after multiple cycles of use, CIP, and regeneration (see Section 3.2). This is particularly important for ensuring the performance and longevity of resin in large-scale manufacturing. Furthermore, the MAM represents a powerful LC-MS-based approach that can be validated not only for identity confirmation but also for comprehensive monitoring of PTMs and other critical quality attributes. By integrating MAM into the analytical workflow, manufacturers can achieve a higher level of process understanding and control, supporting both regulatory compliance and product quality assurance. USP General Chapter <1055> [41] provides detailed guidance on method validation, outlining key parameters including detection limit, specificity, linearity, range, accuracy, precision, and reagent stability. Adhering to these principles ensures that the selected method is robust, reliable, and suitable for its intended purpose within a regulated QC environment.

### 3.2. Application of the Multi-Attribute Method (MAM) for Monitoring Protein A Ligand Modifications

Antibody purification by Protein A chromatography involves sequential steps, i.e., loading, washing, elution, and CIP, which subject the Protein A ligand to variable pH, ionic strength, and chemical stressors. These conditions can trigger PTMs, including oxidation, deamidation, and fragmentation, which compromise ligand integrity and resin performance. Wetterhall et al. from Cytiva [42] applied an LC-MS methodology to evaluate how engineered Protein A ligands withstand acidic and basic wash conditions after amino acid substitutions. In this study, we extend the application of the MAM beyond its conventional use in mAb characterization to Protein A resins, enabling direct analysis of ligand structural changes and their functional impact.

Three pairs of virgin and used resins, MabSelect™ (for MAB1), MabSelect SuRe™ (for MAB2), and MabSelect SuRe™ LX (for MAB3), were evaluated. Used resins were obtained internally and had undergone 116, 291, 112 operational cycles, respectively. All resins were filtered, adjusted to uniform concentrations in the digestion buffer, and injected at consistent volumes to ensure reliable quantification using UV-TPA, as described in Section 2.6.

**Figure 2:**
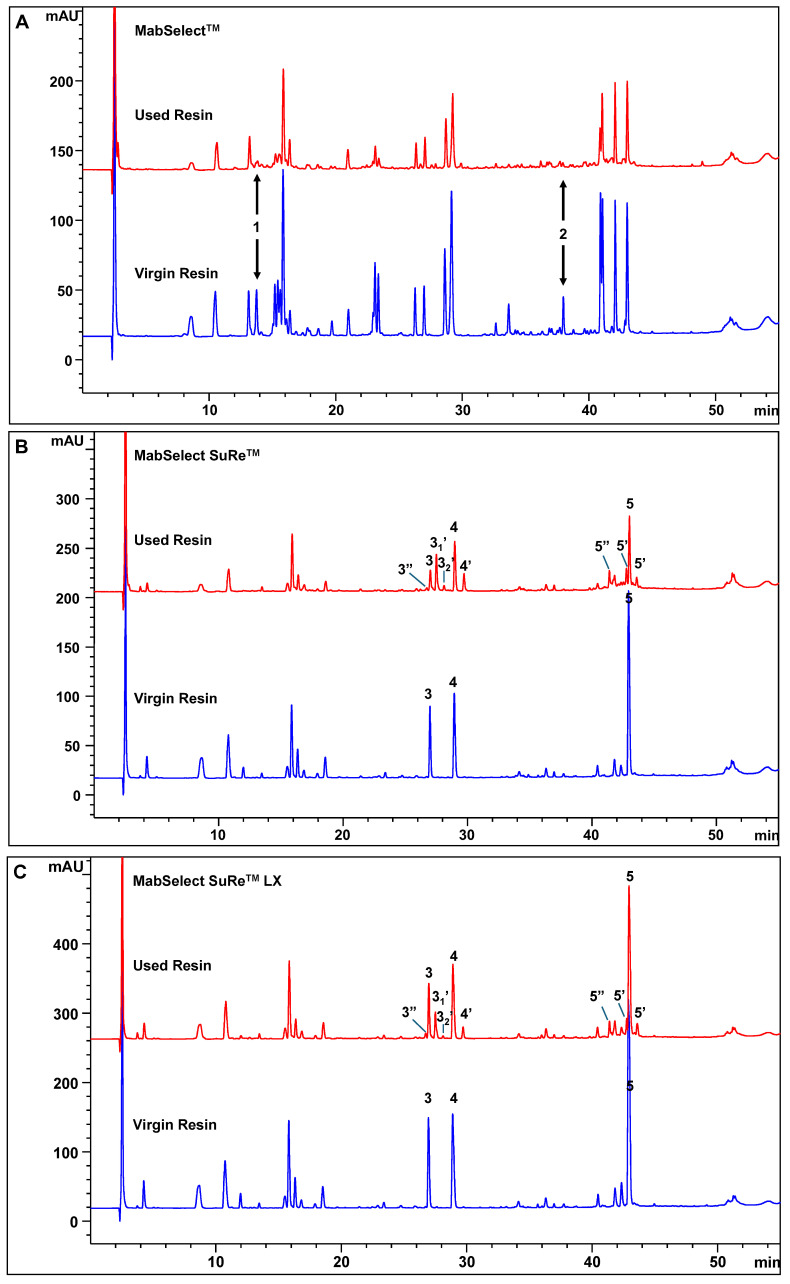
Peptide Mapping Profiles of Virgin and Used Protein A Resins. (**A**) MabSelect™; (**B**) MabSelect SuRe™; (**C**) MabSelect SuRe™ LX. Some modified peaks are labeled as indicated.

LC chromatograms of virgin and used resins subjected to different cleaning cycles are shown in Figure 2. Compared with virgin material, used resins consistently exhibited reduced overall signal intensity. For the MabSelect™ resin, peptides corresponding to residues 1–124 of the wild-type Protein A ligand were largely absent after 116 cycles. Instead, two prominent peaks corresponding to peptides derived from the N- and C-terminal regions of the missing sequence, residues 118–124 (Peak 1) and 1–25 (Peak 2), were observed in virgin resin (Figure 2A). The marked loss of peptides spanning residues 1–124 in the used MabSelect™ resin likely reflects fragmentation pathways induced by repeated exposure to acidic and alkaline cleaning conditions. In addition, proteolytic activity originating from harvested cell culture fluid may further contribute to the cleavage of the Protein A ligand [43].

MAM analysis further revealed substantial chemical modifications in MabSelect SuRe™ and MabSelect SuRe™ LX resins following multiple purification cycles, as summarized in Figure 2B,C, and Table 3. Several peptide sites exhibited asparagine deamidation (Peaks 3′, 4′, and 5′) and aspartic acid isomerization (Peaks 3″ and 5″), both of which are known to be sensitive to pH fluctuations. Analysis of Peak 3 revealed two distinct deamidation events, identified as 3_1_′ (mono-deamidation) and 3_2_′ (double deamidation) at separate sites within the peptide sequence, while Peak 5 displayed two chromatographically resolved species corresponding to isoaspartate (isoD) and aspartate (D), eluting before and after the native Peak 5, respectively (Figure 2B,C). These findings demonstrate that, although engineered Protein A ligands exhibit enhanced resistance to alkaline degradation through targeted amino acid substitutions, they remain susceptible to cumulative chemical modifications during repeated CIP cycles.

### 3.3. Application of the Multi-Attribute Method (MAM) for Monitoring the Dynamic Binding Capacity of Resins

DBC is a critical performance parameter in Protein A affinity chromatography for the purification of therapeutic monoclonal antibodies. It defines the maximum amount of antibody that a resin can bind before breakthrough occurs under specified flow rates and operating conditions. As such, DBC directly influences process efficiency, yield, throughput, and overall cost-effectiveness [44]. In practical terms, DBC serves as a key indicator of resin performance, reflecting the amount of target antibody that can be consistently captured within defined operational constraints. Beyond its analytical significance, DBC is fundamental to process development and manufacturing, as it guides column sizing, cycle scheduling, and productivity optimization. By maximizing DBC within acceptable processing limits, manufacturers can enhance both process efficiency and economic viability across bioprocessing workflows.

**Figure 3:**
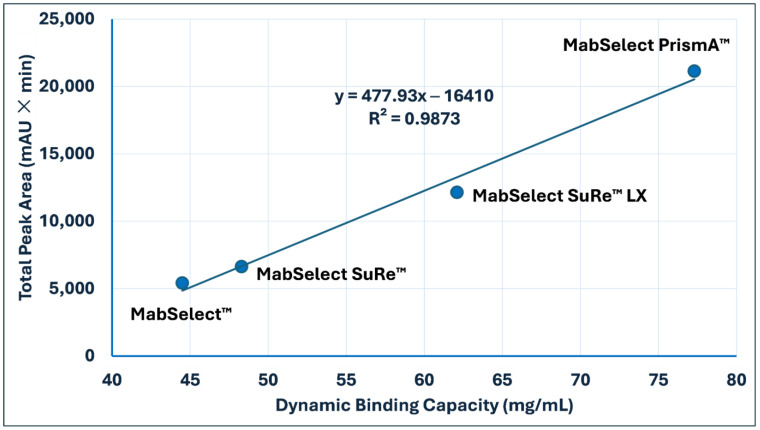
The Correlation between Dynamic Binding (DBC) and the Total Peak Area (TPA) of four Cytiva Resins. TPA values were calculated from the integrated UV signals of the peptide-mapping chromatograms (MAMs) for the four evaluated Protein A resins. The correlation demonstrates the relationship between functional resin capacity and the abundance of ligand-specific peptides.

The DBC of the resins was experimentally determined according to the procedure described in Section 2.5. For virgin resins, the measured DBC values exhibited a strong linear correlation with the total peak area (TPA) obtained from peptide-mapping chromatograms, yielding a correlation coefficient (*r*) of 0.99 (Table 4 and Figure 3). Although these results demonstrate that MAM is applicable not only for resin identity testing but also as a valuable complementary approach for predicting or confirming resin DBC, this correlation should be interpreted as a platform-specific indicator rather than a universal quantitative predictive tool due to the limited dataset. Further studies involving a wider diversity of resin matrices and ligand architectures are necessary to validate the broader applicability of this predictive framework.

For accurate quantitative estimation of DBC based on UV-derived TPA, sufficient proteolytic digestion is critical. Increased enzyme loading is required to ensure complete digestion of resins containing substantially higher ligand densities, such as MabSelect™ PrismA, thereby enabling reliable comparison across different resin platforms. To further evaluate digestion efficiency, the remaining resin material was subjected to a second trypsin digestion under the same conditions. This second digestion did not yield additional significant peptide signals, indicating that the initial digestion was effectively complete and that ligand accessibility within the resin was not a limiting factor.

Applying this methodology to used resins, Table 5 summarizes the measured DBC values and corresponding UV TPA for the three resin types. A progressive decline in DBC was observed with repeated use, accompanied by a reduction in TPA, primarily attributable to Protein A ligand fragmentation and the accumulation of chemical modifications. Repeated exposure to harsh CIP conditions accelerates the structural degradation of the ligand, as reflected in the lower TPA and DBC values of used resins compared with their virgin counterparts. Chemical modifications, including deamidation and isomerization, further compromise ligand integrity, disrupt Fc-binding sites, and reduce antibody capture efficiency.

Collectively, ligand fragmentation and chemical modifications contribute to the observed loss in resin performance and shortened operational lifetime. However, additional fouling mechanisms, such as the accumulation of residual antibodies and host cell proteins, may also influence DBC, resulting in a non-linear relationship between DBC and TPA for used resins. Nevertheless, TPA derived from MAM analysis remains a valuable indicator of resin health and functional integrity during lifecycle monitoring.

### 3.4. Application of the Multi-Attribute Method (MAM) for Detection of Residual Antibodies and Host Cell Proteins on Protein A Resins

HCPs are process-related impurities introduced during recombinant protein manufacturing that may compromise product safety and efficacy [45,46]. Despite low-pH elution and aggressive clean-in-place (CIP) procedures (Section 2.2), residual HCPs and mAb species, including aggregates and fragments, can remain bound to the Protein A ligand and accumulate over repeated chromatography cycles. Ligand fragmentation and PTMs induced by repeated exposure to extreme pH and cleaning agents further promote nonspecific impurity retention. However, routine methods to directly detect and quantify HCPs and residual mAb associated with the resin-bound ligand are lacking.

Conventional assays, including enzyme-linked immunosorbent assay (ELISA) and liquid chromatography-tandem mass spectrometry (LC-MS/MS) [46,47], can only measure impurities released into the Protein A eluate or into extractable fractions. Because LC–MS analysis relies on analytes being solubilized and recoverable, host cell proteins (HCPs) and residual mAbs that remain strongly associated with the Protein A ligand surface may not be captured by conventional workflows. Consequently, standard analyses may not completely reflect the total resin-associated impurity burden, which could influence resin performance over extended use.

Implementing MAM directly on Protein A resin mitigates these challenges by facilitating the precise identification and quantification of residual mAbs and HCPs. To evaluate impurity accumulation, three resin types, i.e., MabSelect™, MabSelect SuRe™, and MabSelect SuRe™ LX, were examined. Consistent with previous protocols in Section 2.6, trypsin-digested samples were subjected to an extended LC gradient (95 min) and a tenfold higher injection volume (50 µL) to improve sensitivity for detecting residual mAbs and HCPs. Both HCPs and residual mAbs were effectively identified directly from the used resins (Table 6).

Residual monoclonal antibody (mAb) and host cell proteins (HCPs) were quantified using an established LC-MS-based HCP method [48], in which peak areas of the three most abundant peptides per protein were compared to a protein standard. In this study, trypsin served both as the proteolytic enzyme for digesting the Protein A ligand, HCPs, and bound mAbs, and as an internal standard. During digestion, trypsin undergoes controlled autolysis, generating reproducible and well-characterized tryptic peptides. These trypsin-derived peptides were used as the normalization reference. Residual mAb levels were calculated by comparing the summed peak areas of the three most abundant mAb peptides to those of trypsin peptides, with the trypsin signal defined as 100%; values exceeding 100% therefore indicate mAb peptide signals greater than the trypsin reference within the same LC-MS analysis. HCP levels are reported in parts per million (ppm), while mAb levels are reported as relative percentages due to their higher abundance.

It is worth noting that the trypsin-referenced “top-peptides” approach used for host cell protein (HCP) measurement is best described as semi-quantitative (relative) rather than fully quantitative. This metric relies on surrogate peptides and is therefore influenced by several variables, including (i) digestion efficiency and peptide recovery, (ii) peptide selection bias and protein-dependent detectability, (iii) peptide-specific ionization efficiency, and (iv) minor instrument drift. To minimize run-to-run variability and ensure consistency, all samples were analyzed within the same LC-MS sequence using an identical amount of trypsin internal standard. This experimental design supports robust relative comparison of HCP and residual mAb trends across resin samples, while avoiding over-interpretation as absolute quantification.

Application of MAM revealed distinct performance differences among the evaluated resins (Table 6). The three predominant HCPs identified from MabSelect^TM^ resin are thrombospondin-1 isoform X2, actin, and elongation factor 1-alpha 1, while no HCPs were detected for the other two resins. Moreover, MabSelect™ showed substantial fouling, with retained MAB1 increasing by 185% after 116 cycles, whereas MabSelect SuRe™ retained only 0.38% MAB2 after 291 cycles, and MabSelect SuRe™ LX retained 18% MAB3 after 112 cycles, indicating improved resistance to impurity accumulation and cleaning efficiency for the engineered resins.

Differences in residual mAb and HCP profiles reflect resin-specific ligand chemistry and cleaning strategies. Resins with native or less chemically robust Protein A ligands require milder CIP conditions, which may limit contaminant removal and increase residual impurities, whereas alkaline-resistant ligands tolerate more aggressive CIP, enabling improved impurity clearance, reduced fouling, and enhanced long-term performance.

Residual mAb levels were also influenced by process-specific loading conditions. Specifically, the MAB2 load concentration applied to the MabSelect SuRe™ column was substantially lower than the MAB1 and MAB3 loads used for the other resins, which likely contributed to the lower residual mAb levels observed for MabSelect SuRe™, even after 291 cycles.

The LC-MS-based MAM approach enables simultaneous quantification of residual mAb and HCPs on Protein A resins and mapping of post-translational modifications after repeated use, improving sensitivity and specificity for process monitoring and troubleshooting.

### 3.5. Application of the Multi-Attribute Method (MAM) to Support Cleaning Strategy Development

CIP practices for Protein A resins are usually determined by vendor guidelines and then fine-tuned through hands-on experiments. Manufacturers offer standard protocols, such as recommendations for sodium hydroxide concentration, contact time, and cycle frequency, which are optimized to balance cleaning effectiveness with ligand stability. Users may further adjust these parameters during process development to target specific product fouling and validate the resin’s lifespan, aiming for thorough cleaning while minimizing ligand loss.

A resin cleaning procedure assessment has been developed for MAB1 using MabSelect^TM^ PrismA, with the objective of determining optimal cleaning parameters to achieve an effective balance among cleanliness, sanitization, and resin longevity. In addition to its increased binding capacity, MabSelect^TM^ PrismA exhibits enhanced resistance to caustic agents compared to the conventional MabSelect^TM^ resin, thereby allowing the use of higher sodium hydroxide concentrations. This enables the implementation of a more rigorous cleaning protocol that may significantly reduce bioburden, minimize resin fouling, and extend the functional lifespan of the resin. The experimental design comprises twenty MabSelect™ PrismA chromatography cycles per column, evaluating six distinct cleaning conditions alongside a control.

**Figure 4:**
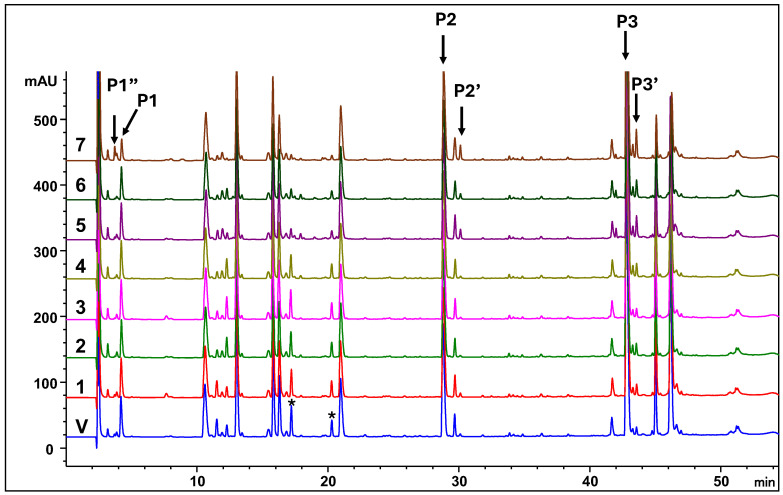
Comparative HPLC-UV Peptide Mapping of MabSelect™ PrismA Resin. Chromatograms display tryptic peptide profiles for (V) virgin resin and (1–7) resin subjected to seven distinct CIP conditions. Original ligand peptides are identified as P1–P3. Post-treatment modifications are highlighted: P1″ denotes the isomerization product of P1, while P2′ and P3′ represent deamidation variants of P2 and P3, respectively, resulting from the specific cleaning strategies. Peaks marked with asterisks (*) are located outside the primary binding region and are not included in the quantitative analysis.

Table 7 summarizes the MAM-based evaluation of MabSelect™ PrismA resin, including residual monoclonal antibody (mAb) levels, HCP clearance, and the extent of ligand modification under different CIP strategies (Table 1). PTMs, specifically aspartic acid isomerization and asparagine deamidation, were visualized for three representative peptides in Figure 4. Residual MAB1 heavy and light chains were consistently detected on all used resins across the seven CIP conditions, but were absent in the virgin resin. Similarly, HCPs were detected under all conditions except for the virgin resin. Overall, HCP levels were low and decreased with increasing sodium hydroxide concentration, with the lowest levels observed under Condition 6, in which CIP was performed after every chromatography cycle. In contrast, PTM levels exhibited an increasing trend with higher sodium hydroxide concentrations. The reported modifications correspond specifically to peptides within the Protein A antibody-binding domain. Although additional peptide variants (examples marked with asterisks “*” in Figure 4) showed variability across CIP conditions, they were excluded from quantitative analysis because they are located outside the primary binding region and are therefore unlikely to affect resin binding capacity.

Data from this study support the use of 0.25 N sodium hydroxide applied every fourth process cycle, as this condition (Condition 4) achieved improved resin cleanliness relative to the control. Although all evaluated conditions outperformed the control, the 0.25 N sodium hydroxide regimen provided the greatest reduction in residual foulants, including MAB1 and host cell proteins, while minimizing PTMs of the Protein A ligand. This indicates an optimal balance between cleaning efficacy and ligand preservation.

In contrast, although 0.25 N sodium hydroxide was also used for CIP in Condition 7, elevated levels of isomerization (P1) and deamidation (P2 and P3) were observed relative to other experimental conditions (Table 7). This effect may be attributed to the higher concentration of the strip solution (1 M acetic acid). High acetic acid concentrations can promote direct hydrolysis of the asparagine side-chain amide, leading to increased deamidation [49]. Additionally, acetate has been reported to catalyze succinimide formation at aspartic acid residues, thereby accelerating isomerization [50].

This study represents the first application of a MAM LC–MS strategy to comprehensively assess Protein A resin integrity. By simultaneously quantifying residual foulants (mAbs and HCPs) and site-specific PTMs of the ligand, this approach offers a level of granularity unattainable by legacy methods. Whereas previous investigations relied heavily on gel-based assays, which often depend on subjective visual interpretation of subtle fouling patterns, our MAM-based framework provides a sensitive, high-resolution alternative for evaluating resin degradation.

## 4. Conclusions

This study represents the first comprehensive application of the MAM to characterize Protein A affinity resins throughout their operational lifecycle. While previous research has used LC-MS for isolated investigations of resin fouling or dynamic binding, the primary innovation of this work lies in the development of a single, integrated MAM workflow that simultaneously tracks resin identity, ligand PTMs, residual impurities, and functional activity. By enabling molecular-level interrogation of Protein A ligand identity, structural integrity, and PTMs, MAM provides mechanistic insights into resin aging and degradation pathways that are not accessible through conventional qualification and performance assays alone. In contrast to traditional bulk metrics, MAM directly links molecular alterations, such as ligand fragmentation, chemical modification, and impurity accumulation, to functional performance outcomes in a single analytical platform.

When integrated with (DBC measurements, which serve as a primary indicator of resin functionality, MAM elucidates how progressive molecular changes induced by repeated use and CIP exposure can precede, explain, and ultimately contribute to declines in binding performance. This combined analytical strategy enables early detection of ligand damage and fouling mechanisms prior to substantial loss of capacity, offering a predictive framework for monitoring resin health and performance over time.

The integration of MAM with conventional performance metrics establishes a more holistic and mechanistically informed paradigm for Protein A resin lifecycle management. This approach enables data-driven optimization of resin selection, rational design of CIP strategies, and scientifically justified determination of resin end-of-life. Moreover, it supports improved risk assessment, enhanced process understanding, and proactive maintenance of purification performance.

As next-generation Protein A resins continue to evolve toward higher binding capacities, increased ligand densities, and enhanced alkaline stability, advanced analytical tools capable of resolving subtle molecular changes become increasingly critical. The incorporation of MAM provides a forward-looking, scalable analytical framework to strengthen process robustness, ensure purification consistency, and enhance quality assurance in mAb manufacturing. Ultimately, this methodology supports the development of more resilient, efficient, and sustainable downstream bioprocesses.

## Figures and Tables

**Table 1:** The Processing Conditions for Evaluating Resin Cleanability and Lifecycle.

Condition	Condition 1	Condition 2	Condition 3	Condition 4	Condition 5	Condition 6	Condition 7
Strip Solution	1% Acetic Acid, 1.17% Phosphoric Acid					N/A	1 M Acetic Acid
Strip Hold	1 h	1 h	N/A	N/A	N/A	N/A	N/A
WFI Flush CV(s)	2	1	1	1	1	1	1
Displace Buffer	100 mM Tris pH 7	N/A					
Displace CV(s)	0.5	N/A					
CIP Solution 1 *	0.05 N NaOH, 1 M NaCl	0.1 N NaOH	0.1 N NaOH	0.25 N NaOH	0.5 N NaOH	0.5 N NaOH	0.25 N NaOH
CV(s)/Contact Time	2 CV/10–20 min	2 CV/30 min	2 CV/30 min	2 CV/30 min	2 CV/30 min	2 CV/15 min	2 CV/30 min
Displace Buffer	100 mM Tris pH 7	WFI					
Displace CV(s)	1 CV						
CIP Solution 2 *	N/A	1 M Acetic Acid					
CV(s)/Contact Time	N/A	2 CV/30 min	2 CV/30 min	2 CV/30 min	2 CV/30 min	2 CV/30 min	2 CV/30 min
Displace Buffer	100 mM Tris pH 7	WFI					
Displace CV(s)	1 CV						

Abbreviation. CV: column volume, CIP: Clean-in-Place. WFI: Water for Injection. NaOH: sodium hydroxide. * CIP was performed on every fourth cycle for Conditions 1–5, and 7, while CIP was performed every cycle for Condition 6.

**Table 2:** Mass-to-Charge (*m*/*z*) and Retention Times of Peptides from Different Protein A Resins.

Resin Name	Blue Box		Yellow Box	
	Retention Time (min)	MS *m*/*z*	Retention Time (min)	MS *m*/*z*
MabSelect™ PrismA	20.985	715.432	28.838	811.067
MabSelect SuRe™ LX	N/A	N/A	28.878	825.075
MabSelect SuRe™	N/A	N/A	28.939	825.075
MabSelect™	21.011	773.437	29.143	825.075

**Table 3:** Post-Translational Modifications (PTMs) of Virgin and Used MabSelect SuRe™ and MabSelect SuRe™ LX Resins.

Original Peptide Peak	Modified Peptide Peak	Modification	MabSelect SuRe^TM^ (MAB2)		MabSelect SuRe^TM^ LX (MAB3)	
			Virgin	Used	Virgin	Used
3	3′ (3_1_′ + 3_2_′)	Deamidation	1.6	45.9	N/D	27.8
	3”	Isomerization	1.3	9.7	1.4	10.0
4	4′	Deamidation	N/D	15.7	N/D	7.0
5	5′	Deamidation	2.2	23.9	2.2	11.2
	5″	Isomerization	0.1	19.5	0.1	11.3

**Table 4:** The DBC and Total Peak Area (TPA) of four Virgin Cytiva Resins.

Resin Name	Measured DBC (mg/mL)	Total Peak Area (mAU × Min)
MabSelect™ PrismA	77.3	21143
MabSelect SuRe™ LX	62.1	12145
MabSelect SuRe™	48.3	6633
MabSelect™	44.5	5417

**Table 5:** The Dynamic Binding Capacity (DBC) and HPLC Total Peak Area (TPA) of Used Resins.

Resin Name	Chromatography Cycles	Measured DBC (mg/mL)	Total Peak Area (mAU × Min)
MabSelect™	116	31.8	3201
MabSelect SuRe™	291	30.5	4823
MabSelect SuRe™ LX	112	24.8	9848

**Table 6:** The Residual mAb and HCPs from Three Types of Used Resins.

Resin Name	MabSelect™ (MAB1)	MabSelect SuRe™ (MAB2)	MabSelect SuRe™ LX (MAB3)
Chromatography Cycles	116	291	112
Total mAb (%)	185%	0.38%	18%
Totals HCPs (ppm)	272,610	N/D	N/D

N/D: Not Detected.

**Table 7:** Impact of CIP Strategies on MabSelect™ PrismA Performance and Ligand Integrity.

		Conditions (Refer to Table 1)							
		Virgin	1 *	2	3	4	5	6	7
UV-TPA		21,886	22,042	20,036	20,276	19,043	20,002	17,440	16,952
Residual mAb (%)		0	3.0	2.0	1.6	1.7	1.1	0.6	1.6
Total HCPs (ppm)		0	17,734	4040	2465	395	366	71	1039
Peptide Modifications	P1” Isomerization	9.5	9.8	5.2	5.5	5.3	12.5	8.1	36.4
	P2′ Deamidation	2.6	3.7	2.2	4.4	4.6	7	6.2	9.6
	P3′ Deamidation	0.3	0.4	0.3	0.6	0.8	3.6	3.1	1.7

* condition 1 is the control condition.

## Data Availability

All data supporting the findings of this study are available from the corresponding authors upon request.

## Abbreviations

Abbreviation	Definition
CIP	Clean-in-Place
DBC	Dynamic Binding Capacity
HCP	Host Cell Protein
LC-MS	Liquid Chromatography–Mass Spectrometry
mAb	Monoclonal Antibody
MAM	Multi-Attribute Method
PTM	Post-Translational Modification
TFA	Trifluoroacetic Acid
TPA	Total Peak Area
